# Taxonomy of Individual Variations in Aesthetic Responses to Fractal Patterns

**DOI:** 10.3389/fnhum.2016.00350

**Published:** 2016-07-08

**Authors:** Branka Spehar, Nicholas Walker, Richard P. Taylor

**Affiliations:** ^1^School of Psychology, UNSW AustraliaSydney, NSW, Australia; ^2^Department of Physics, University of OregonEugene, OR, USA

**Keywords:** individual differences, fractals, visual preference, aesthetics, individual variability

## Abstract

In two experiments, we investigate group and individual preferences in a range of different types of patterns with varying fractal-like scaling characteristics. In Experiment 1, we used 1/f filtered grayscale images as well as their thresholded (black and white) and edges only counterparts. Separate groups of observers viewed different types of images varying in slope of their amplitude spectra. Although with each image type, the groups exhibited the “universal” pattern of preference for intermediate amplitude spectrum slopes, we identified 4 distinct sub-groups in each case. Sub-group 1 exhibited a typical peak preference for intermediate amplitude spectrum slopes (“intermediate”; approx. 50%); sub-group 2 exhibited a linear increase in preference with increasing amplitude spectrum slope (“smooth”; approx. 20%), while sub-group 3 exhibited a linear decrease in preference as a function of the amplitude spectrum slope (“sharp”; approx. 20%). Sub-group 4 revealed no significant preference (“other”; approx. 10%). In Experiment 2, we extended the range of different image types and investigated preferences within the same observers. We replicate the results of our first experiment and show that individual participants exhibit stable patterns of preference across a wide range of image types. In both experiments, Q-mode factor analysis identified two principal factors that were able to explain more than 80% of interindividual variations in preference across all types of images, suggesting a highly similar dimensional structure of interindividual variations in preference for fractal-like scaling characteristics.

## Introduction

Dating back to the Greek philosophers from the 5th century BC, the attempts to understand aesthetics can be cast as a continuous debate between views that consider it determined by objective properties of objects, vs. those that emphasise subjective characteristics of observers in aesthetic appreciation. Both the notions of the universal canons of beauty on the one hand and the beauty as in the eye of the beholder on the other, have been and remain widespread reflection of these opposing views.

Launched by Fechner (1876), experimental aesthetics was an attempt to empirically ground the aesthetic experience as coming from “below”, rather than being a quality of ever-changing individual and/or cultural and philosophical contexts (Fechner, 1876). Early incarnations of this approach included the strict mathematical proportions used in ancient architecture, most famously those concerning proportion and symmetry. Fechner’s (1876) own investigations focused on the golden ratio and he found that observers when asked to choose the most preferred from the series of 10 rectangles varying in aspect ratio, preferred the rectangle with proportions corresponding to the golden ratio. While Fechner’s (1876) findings have been criticized on methodological grounds (McManus, 1980; Russell, 2000), subsequent investigations of aesthetic preference in nearly all domains have continually sought, and succeeded, in demonstrating robust universal preferences for image properties ranging from balance (Arnheim, 1974; McManus et al., 2010), fractal dimension (Aks and Sprott, 1996; Spehar et al., 2003, 2015), to symmetry (Bertamini et al., 2013), informational content and complexity (Eysenck, 1941; Berlyne, 1971; Garner, 1974), curvature (Hogarth, 1753; Bar and Neta, 2006; Gómez-Puerto et al., 2016) as well as contrast and clarity (Gombrich, 1979).

However, while numerous studies have verified consistent aesthetic preference for certain stimulus properties, there has been a certain reluctance to use these results to claim the universal nature of their aesthetic appeal. Indeed, nearly a century ago, Thorndike (1917) was among the first to point out that “the average tendency towards this or that preference may give an impression of greater uniformity than exists”, and concluded that although any one person usually feels very decided with strong preferences, these are rarely shared by others.

Thorndike’s (1917) study was motivated by Fechner’s (1876) claims regarding the preference for golden ratio. He used a number of different classes of stimuli (rectangles, triangles, crosses, and lines) varying in proportion and asked observers to first choose one item in each series that they liked the look of most, then next most, and so on. Although Thorndike (1917) found that the most liked rectangle and triangle had the ratio of height to base which was close to the golden ratio (1.8:1 for rectangles and 1.7:1 for triangles respectively), the rankings given by different observers varied considerably and even those most liked were ranked the lowest by some observers. Thorndike’s (1917) initial observations were later echoed by a number of subsequent studies (McManus, 1980; Hoge, 1995; Höfel and Jacobsen, 2003; McManus et al., 2010), confirming that the universal preference for the golden ratio is weak and that it might in fact not exist if one uses bias-free methodology in measuring aesthetic preferences.

In particular, McManus (1980) and McManus et al. (2010) used the method of paired comparison, singled out as a particularly good methodological option in measuring aesthetic preference, to measure the preference for rectangles varying in aspect ratio. They found that although population or group preference for golden section was weak, the individual rectangle preference functions were strong and stable over time but highly variable between different participants. In order to characterize the highly variable patterns of rectangle preferences seen at an individual level, McManus et al. (2010) analyzed the structure of the differences using a Q-mode factor analysis. Q-mode analysis revealed two distinct factors: one that is loaded with preferences for rectangles close to square, and the other loaded with preferences for elongated rectangles. Together they explained 44% of variability and were also stable over time as indicated by retests conducted up to months, or even years later. Interestingly, the differences in rectangle preference functions did not correlate with any of the Big Five personality traits, or a number of other individual difference measured ranging from need for cognition, tolerance of ambiguity and schizotypy, to vocational types, and aesthetic activities. Indeed, at present, the large interindividual differences in the rectangle preference functions remain unexplained (McManus et al., 2010).

In another influential study of interindividual differences in aesthetic preference, Vessel and Rubin (2010) looked at relative preference between pairs of stimuli for a large sample of real-world and abstract images. Vessel and Rubin (2010) also found that while individual observers displayed robust and consistent preferences, the agreement between observers was quite low. Importantly, the interindividual agreement between different observers was much higher in preference for real world compared to abstract images. In order to explain this pattern of results, Vessel and Rubin (2010) have strongly emphasized the role of factors internal to individual observers and observers’ personal experiences as critical in contributing to preference. They argue that visual preferences are not driven by the physical attributes of these stimuli *per se*, but by the semantic content of these stimuli. The high agreement in real-world images is attributed to common experiences and shared semantic associations with visual stimuli, with shared semantic interpretations leading to the shared preferences as well. Abstract images contain more ambiguity and evoke different meaning for different observers, leading to the low level of interindividual agreement. These notions were echoed and further developed in Vessel et al. (2012) claims that individuals’ taste in art is unique and intertwined with their “sense of identity” and personal relevance.

In summary, there is growing evidence that interindividual differences play an important role in preference functions for a range of different images and we are only beginning to broaden our understanding of the structure and causes of these variations.

## Experiment 1: Group and Individual Preferences for Patterns Varying in Fractal Scaling Characteristics

In the present study, we focus on the individual differences in visual preference for fractal patterns. A number of studies have revealed robust average preference for mid-range fractal dimension across many different types of fractal patterns including geometrical shapes (Aks and Sprott, 1996); abstract paintings, natural and computer generated fractal patterns (Spehar et al., 2003); horizon lines (Hagerhall et al., 2008); and synthetic images with fractal-like scaling characteristics (Juricevic et al., 2010; Spehar and Taylor, 2013). These highly consistent preferences, observed despite the wide range of stimuli and methods used in these studies, suggest that certain fractal characteristics might be an objective image property that is universally linked with aesthetic preference (see also Redies, 2007, 2015). Spehar et al. (2015) have also demonstrated a close link between visual preference and visual system’s ability to detect and discriminate amongst the patterns with fractal-like scaling.

However, the degree of interobserver agreement or disagreement in these studies was not investigated, and the extent of inter-observer agreement in preference for fractal patterns remains unknown. Thus, the main purpose of this study is to further probe the characteristics of both population and individual preference functions for variations in fractal characteristics. We investigate preference in different types of fractal patterns in order to capture the variability of fractal images used in previous studies and to be able to directly compare patterns of group and individual preferences over a wider range of images than has been done before.

**Figure 1:**
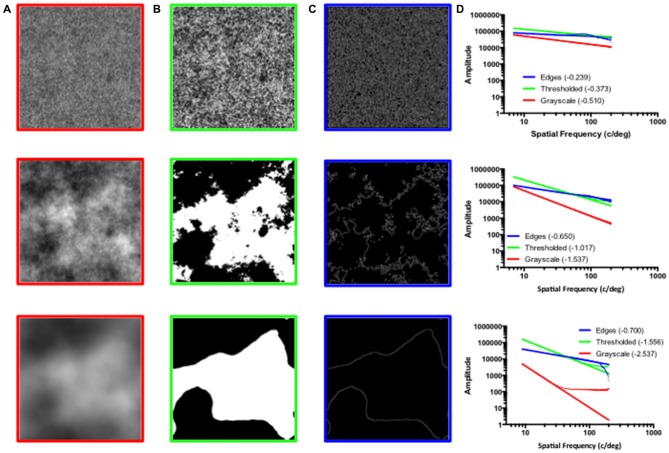
**(A)** Grayscale fractal patterns with exponent values of 0.5 (top), 1.5 (middle), and 2.5 (bottom). **(B)** Black and white fractal patterns created by thresholding the grayscale patterns from the panel **(A)** at their mean luminance level. **(C)** Edges only patterns depicting the fractal boundaries created by extracting edges from the thresholded patterns depicted in the panel **(B)**. **(D)** The amplitude spectra plots corresponding to the grayscale (red), thresholded (green) and edges only (blue) patterns; *x*-axis represents log spatial frequency while *y*-axis represents log amplitude. The lines (bold) are linear fits to the data (light).

In line with most of the previous studies, we use “statistical” as opposed to the “exact” fractals. Whereas exact fractals contain exact repetitions of a certain pattern at different magnifications, statistical fractals contain a certain degree of randomness that disrupts the precise repetition so they only appear similar at different magnifications. The three types of patterns used in this study are illustrated in Figure 1. The first column represents synthetic noise images generated using a 1/f^α^ distribution of rotationally averaged spatial scale specific amplitude variations. From top to bottom, the synthetic images are increasing in the exponent alpha, slope of the best fitting linear function applied to the rotationally averaged amplitude spectrum. Plotted in the second column are the binary variants of grayscale patterns, generated by thresholding the grayscale image at the mean luminance level, such that pixels below mean luminance are assigned as white and those above are black. Finally, the images in the third column were generated by extracting edges from the black and white image in the middle column. Conventionally, the fractal-like variations in the grayscale images are referred to as “two dimensional” (2D) fractals, because they form surface textures, whereas the thresholded and edge patterns are referred to as “one-dimensional (1D) fractals because they form fractal lines between the black and white regions.

It is important to emphasise that whereas the input 1/f alpha slope values used to generate stimuli across the three corresponding image types are identical, the thresholding and edge extracting procedures alter the measured amplitude spectrum slope values. In general, the thresholded and edges only images result in a shallower 1/f slope, particularly at steeper amplitude spectra slope values. This relationship can be seen in the amplitude spectra plots depicted in the right most column of Figure 1.

**Figure 2:**
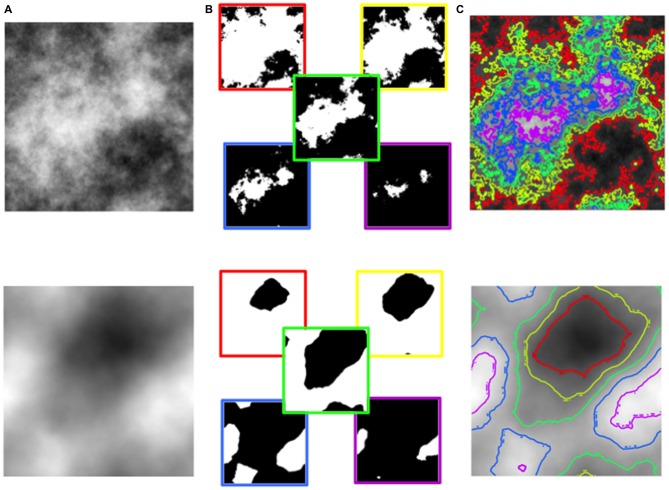
**(A)** The original grayscale synthetic noise image with the 1/f slope of −1.5 (top) and −2.0 (bottom). **(B)** The thresholded variants of the grayscale images thresholded at relative luminance values of 0.1 (red), 0.3 (yellow), 0.5 (green), 0.7 (blue) and 0.9 (purple). **(C)** The contours extracted at the respective thresholded luminance values superimposed on the original grayscale image. The average fractal dimension of the contours in the top image equals 1.41 (ranging from 1.38 to 1.43) and 1.13 (ranging from 1.05 to 1.18) in the bottom image.

However, while the photometric properties (amplitude spectra) between grayscale and thresholded image types differ, their geometric or fractal-scaling properties are nearly identical (Spehar and Taylor, 2013). That these three types of images share their geometric, fractal scaling properties, despite their visually distinct visual appearances, can be demonstrated by thresholding the grayscale images at multiple luminance levels and examining the geometrical characteristics of the resulting thresholded and edge only images (Spehar and Taylor, 2013). This is illustrated in Figure 2 with two grayscale patterns shown on the left (panel A), which have been thresholded at five different luminance levels, as shown in the middle (panel B). Although it might not seem intuitive at first, the edges visible at the boundaries between black and white regions in different thresholded variants have nearly identical fractal scaling characteristics, as is evident from the contour graph superimposed on the original grayscale images, as depicted on the right (panel C). Here, the differently colored edges correspond to those extracted at particular thresholded levels. One can immediately discern that in both top and the bottom panels, the edges extracted at different thresholded luminance levels are nested within each other and appear similar in their spatial appearance. Moreover, it is obvious that the edges extracted in this way from the grayscale image with a shallower amplitude spectrum slope (bottom image) have smoother and less jagged geometrical characteristics, consistent with different fractal scaling characteristics associated with these images. Together, these demonstrations show how the same contour-based fractal characteristics can be traced even to the grayscale images that do not contain explicitly visible sharp boundaries due to the continuous intensity variations. As these demonstrations show, the grayscale images can nevertheless be conceived as a series of nested boundary and contour patterns at different threshold intensities. Thus, all three classes of images share the shape and geometrical characteristics of their either explicit or implicit boundary contours, determined by the amplitude spectrum of grayscale images, as demonstrated in Figure 2.

The shape of these boundaries can be quantified by a scaling parameter known as the fractal dimension (D), and widely used procedure to determine the fractal dimension of complex patterns is the box-counting technique. While the box-counting is not the only technique by which the fractal dimension can be estimated, it is well-suited to calculating fractal dimension for statistically self-similar patterns such as the grayscale images used in our study (Lopes and Betrouni, 2009). The box-counting technique analyses a 2D image by first converting it into a binary image and then covering it with a mesh of identical spatial segments (“boxes”) of identical length L, and simply counting the number of squares that contain the pattern variations (N). This count is then repeated for increasingly smaller squares, a procedure essentially equivalent to examining the image at finer spatial scales (or frequencies). The scale-invariance of the fractal pattern appears through the power law relationship N ~ (1/L)^D^ (where the exponent D is the fractal dimension). The relationship between the amplitude spectrum slope of the 2D luminance (grayscale) image and the D values is always an inverse, linear relationship, so that an image quantified by an intermediate amplitude spectrum slope value α will also be quantified by an intermediate fractal dimension D value (Knill et al., 1990; Graham and Field, 2007; Fairbanks and Taylor, 2011; Spehar and Taylor, 2013). This relationship is preserved when the 2D luminance images are thresholded at different levels, as illustrated in Figure 2.

Fractal dimension is also a statistical measure of a pattern’s structural complexity, indexing how detail in a fractal pattern changes with the spatial scale. It reflects the relative amount of coarse and fine structure in the pattern, such that patterns with higher amounts of fine structure, and thus higher visual complexity, are quantified by higher D values. Unlike previous measures of complexity, the fractal dimension affords a precise quantification of visual complexity that can be applied across a wide range of visually distinct images. Findings that the visual preference is highest for patterns with intermediate fractal exponents dovetails nicely with the findings that patterns with a moderate degree of “complexity” are consistently preferred to those with higher or lower degrees of complexity (Fechner, 1876; Berlyne, 1971; Nadal, 2007; Forsythe et al., 2011). While it has already been established that the apparent complexity of fractal contours is positively correlated with the value of D (Cutting and Garvin, 1987), the relationship between apparent complexity, fractal dimension and amplitude spectrum slope of synthetic noise images has not been investigated.

We capitalize on the regular relationship between amplitude spectrum slope of synthetic grayscale noise images and fractal dimension for the purpose of investigating observers’ generic visual preferences across a wide range of patterns. In Experiment 1, we investigate both individual and group visual preference in grayscale, thresholded and edges only images, which despite superficial differences in their visual appearance possess similar fractal scaling properties. In a separate group of observers we also investigate perceived complexity of the same patterns. In particular, we aim to establish whether observers’ preferences are determined by the fractal scaling properties or by the exact photometric characteristics of these images. We also investigate the relationship between visual preference and perceived complexity in these patterns.

### Experiment 1: Materials and Methods

#### Participants

A total of 310 Psychology undergraduate students at University of New South Wales (mean age 22.52; 31% male) participated in this experiment in exchange for course credit. Informed consent, testing and debriefing procedures were approved by the UNSW Human Research Ethics Advisory Panel. The preferences among grayscale, thresholded and edges only images were measured in 278 observers, with different observers viewing different images: 94 observers viewed the grayscale images while 95 and 89 observers viewed theresholded and edges only images respectively. An additional 32 observers rated the grayscale, thresholded and edges only images for perceived complexity.

#### Design

This study employed a 3 (fractal image type: grayscale, thresholded, or edges only) × 9 (amplitude spectrum slope: from 0.5 to 2.5 in increments of 0.25) mixed design. Fractal image type was between-subject factor while the amplitude spectrum slope was within-subject factor.

#### Stimuli

##### Grayscale Synthetic Noise Images

**Figure 3:**
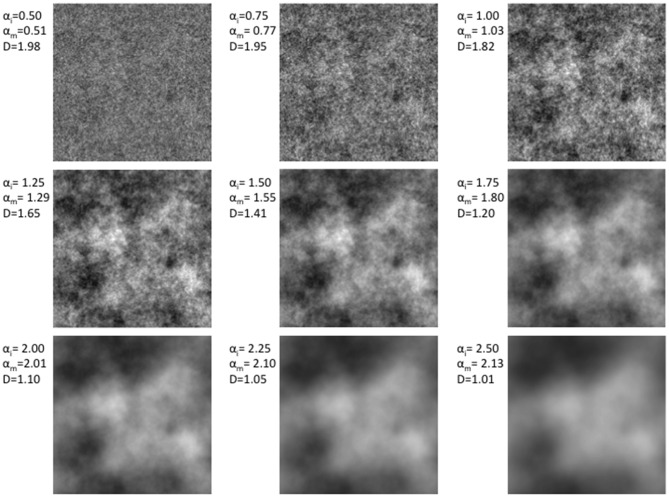
**Grayscale images varying in amplitude spectrum slope from 0.5 to 2.5 in increments of 0.25.** The values next to each image show its respective amplitude spectrum slope used to create the grayscale image (top); its measure amplitude spectrum slope (middle) and its fractal dimension D determined by a box-counting procedure applied to the equivalent edge only images.

The grayscale images were constructed by first creating a 512 × 512 pixels, random noise pattern with each pixel value (0–255) selected from a Gaussian distribution. A Fourier transform was then performed to obtain the amplitude frequency spectrum, which was adjusted to create a range of spectral slopes ranging from 0.5 to 2.5 in increments of 0.25. The nine resulting experimental images had amplitude spectrum slope α values of 0.5, 0.75, 1.00, 1.25, 1.50, 1.75, 2.00, 2.25 and 2.50 and are depicted in Figure 3. Next to each image are the values representing its input amplitude spectrum slope, its measured amplitude spectrum slope and the fractal dimension D calculated by a box-counting procedure. The slopes of amplitude spectra were measured using the *imspect* image processing Matlab function, available through the Standford VISTA experimental control and display toolbox^1^. The number of frequency bins used was the default value of 100. The low cutoff was kept at 2% (also a default value) to avoid the amplitude spikes at low frequencies.

The mean brightness and the RMS contrast of grayscale images were controlled at 126 and 0.30 respectively. Following these specifications, three different sets of seed grasycale images were created, resulting in a total of 27 grayscale noise images (3 seed images × 9 amplitude spectrum slope values).

##### Thresholded Black and White Images

**Figure 4:**
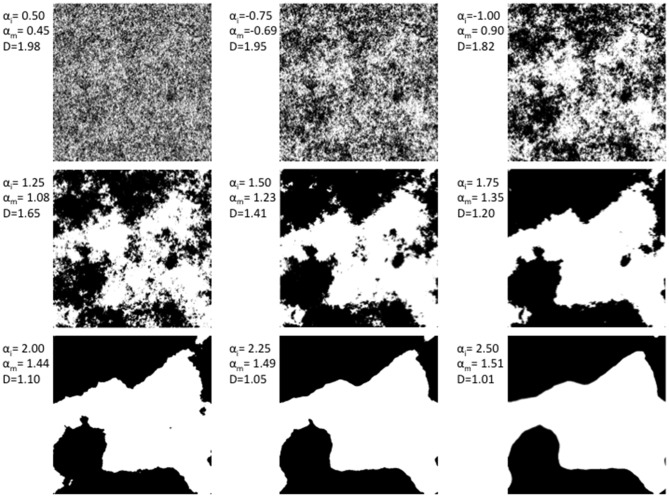
**Thresholded images varying in amplitude spectrum slope from 0.5 to 2.5 in increments of 0.25.** The values next to each image show its respective amplitude spectrum slope used to create the original grayscale image (top); its measure amplitude spectrum slope (middle) and its fractal dimension D determined by a box-counting procedure applied to the equivalent edge only images.

The binary black and white variants of grayscale patterns were generated by thresholding the grayscale image at the mean luminance level, such that pixels below mean luminance were assigned as black and those above as white. The examples of black and white images are illustrated in Figure 4 with the corresponding input and measured amplitude spectrum slope, and fractal dimension D values respectively. As discussed previously, the thresholding process flattens the amplitude spectrum of each image resulting in measured amplitude spectrum slopes that are considerably lower compared to those of the original grayscale images.

##### Edges Only Images

**Figure 5:**
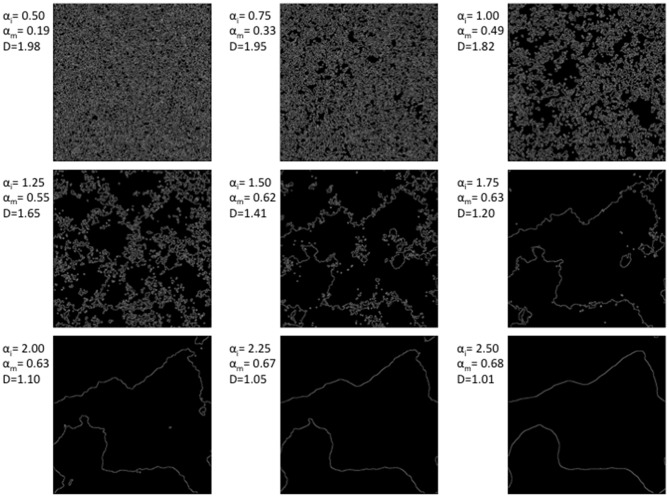
**Edges only images varying in amplitude spectrum slope from 0.5 to 2.5 in increments of 0.25.** The values next to each image show its respective amplitude spectrum slope used to create the original grayscale image (top); its measure amplitude spectrum slope (middle) and its fractal dimension D determined by a box-counting procedure.

Edges only images were created by the Laplacian of Gaussian edge extraction method from the thresholded binary images and are depicted in Figure 5.

#### Apparatus

Testing was done on a Hewlett-Packard workstation, connected to a BenQ 24″ monitor set at its native resolution of 1920 × 1080. The luminance output was linearized and a mean luminance of 58 cd/m^2^ was maintained throughout the duration of all trials in an otherwise dark environment.

#### Procedure

##### Visual Preference Measurements

To investigate preference function in the three classes of images a forced-choice paired-comparison procedure was used. In this task the participants are only required to compare the two images presented at each trial, without needing to pay attention to any stimuli that appeared on previous trials, or anticipate any subsequent stimuli/trials. The participants indicate which image in a pair they prefer by a button press.

For the three image types, each amplitude slope value was paired with all other three amplitude slope values from the same image type, creating 72 unique pairs of grayscale, thresholded and edges only images. Across each set of 72 unique pairs, each pattern is shown an equal number of times overall and an equal number of times on the left and the right side. Each amplitude slope pairing was repeated three times, generating a total of 216 trials that were presented in a random order. For each repetition, images for a different seed image set were used, so that there were no exact repetitions of any trials.

Participants were seated at a viewing distance of 60 cm, with the head stabilized in a height-adjustable chinrest. Each pattern was centered at an eccentricity of 4° and subtended a visual angle of 6°. All stimuli were presented against the uniform gray background of the same luminance. Each trial started with a 500 ms fixation point, followed by the side-by-side stimulus choice display. The task of the observers was to simply indicate (via key press) which of the two stimuli they visually prefer. The duration of the response interval was unlimited.

##### Perceived Complexity Measurements

The same procedure was used to investigate the perceived complexity of the grayscale, thresholded and edges only images. Here, the task of the observers was to indicate (via key press) which of the two stimuli appears more complex.

### Experiment 1: Results and Discussion

#### Average (Group) Preferences for Different Types of Images

**Figure 6:**
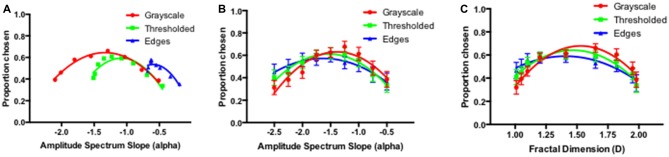
**Average preference for variations in amplitude spectrum slope values in grayscale (red), thresholded (green) and edges only (blue) images plotted as a function of their measured amplitude spectra (A); the amplitude spectra of the input grayscale image (B) and their fractal dimension D, measured by box-counting (C)**.

The raw proportion data have been scaled using (Thurstone, 1927, 1929) approach by converting the individual raw proportion scores into standardized, *z*-scores and the corresponding inverse Gaussian cumulative density function values. The scaled average visual preference functions for grayscale, thresholded and edges only images are depicted in Figure 6. The various panels show the proportion of trials by which the certain image was chosen against all others in a given image type. The error bars correspond to 95% confidence intervals (CI) associated with the respective condition means. Given the variety of images used, the average preferences are plotted in three different ways: (1) as a function of their measured amplitude spectrum slope values (panel A); (2) as a function of the input amplitude spectrum slope of the grayscale seed images from which they were derived (panel B); and (3) as a function of their fractal dimension D (panel C).

As indicated in Figures 3–5, the grayscale, thresholded and edges only images possess different spatial frequency and associated amplitude spectrum characteristics. The amplitude spectrum slope values ranged from −0.68 to −0.19 for edges only images, from −1.51 to −0.45 for the binary thresholded images, and from −2.13 to −0.51 for the grayscale images. If the visual preference is determined by the photometric characteristics of the entire amplitude spectrum of these images, then the preference should be a monotonic function of alpha slope values, irrespective of the image type. It is obvious that the results do not follow this trend and that instead, for each different image type the preferences peak for the intermediate slope values within each range of slope values, regardless of their absolute alpha slope values. That the preference functions for the three different image types are closely aligned is more obvious when they are plotted as a function of either the input alpha of the grayscale seed images from which the thresholded and edge variants were derived (panel B) or the fractal dimension of these images (panel C).

The average preference functions for the three different image types show a similar pattern with the visual preference highest for the intermediate amplitude spectrum slopes or fractal dimension values. Mixed-measures analysis of variance (ANOVA) revealed no main effect of the image type (*F*_(2)_ = 2.34, *p* < 0.100) but a significant main effect of the amplitude slope/fractal dimension (*F*_(8)_ = 28.09, *p* < 0.001) and significant interaction between amplitude slope/fractal dimension and image type (*F*_(16)_ = 2.83, *p* < 0.018). To further explore the average preference functions we performed *post hoc* Holm-Sidak multiple comparison tests between each image type at each of the amplitude spectrum slope values. The number of comparisons per family was three, with nine families, with the significance level set at 0.05. Out of 27 comparisons in total, only five were statistically significant. The *post hoc* comparisons revealed significant differences between grayscale and edges only images at amplitude spectrum slope values of −1.0 (*t* = 2.678, *p* < 0.05), −1.25 (*t* = 3.536, *p* < 0.01), −2.0 (2.699, *p* < 0.05), −2.25 (*t* = 3.075, *p* < 0.05) and −2.5 (*t* = 3.913, *p* < 0.01). Interestingly, the difference between thresholded and grayscale images and thresholded and edges only images do not reach significance at any of the input amplitude spectrum slope (or fractal dimension) values. This pattern is consistent with Spehar and Taylor (2013) who separately compared grayscale and thresholded images and thresholded and edges only images and found no difference between different image type in either of the comparisons.

**Figure 7:**
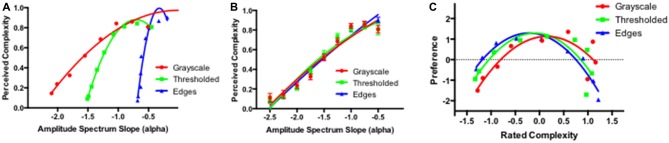
**Average perceived complexity in grayscale, thresholded and edges only images plotted as a function of their measured amplitude spectra (A)**; and the amplitude spectra of the input grayscale image **(B)**. **(C)** Average preference for the three types of images as a function of perceived complexity of these images.

The perceived complexity of the grayscale, thresholded and edges only images are shown in Figure 7, plotted as a function of the measured amplitude spectrum slope of these images (panel A), and as a function of the amplitude spectrum slope of the grayscale seed images (panel B). Similar to what was observed with the visual preference data, the ratings of perceived complexity as a function of the measured slope of the three types of images do not follow an overall monotonic trend. Instead, the perceived complexity increases almost linearly within the each range of amplitude spectrum slope values for the three image types. In all cases, the steeper amplitude spectrum values are associated with lower ratings of complexity compared to the images in the same class but with the shallower amplitude spectrum slope values. When plotted as a function of the amplitude spectrum slope of the input grayscale seed images, the ratings of complexity for the grayscale, thresholded and edges only images exhibit a nearly perfect overlap (panel B). Mixed-measures ANOVA revealed no main effect of image type (*F*_(2)_ = 1.76, *p* < 0.178) but a significant main effect of the amplitude slope/fractal dimension (*F*_(8)_ = 1260, *p* < 0.00001) and a significant interaction between amplitude slope/fractal dimension and image type (*F*_(16)_ = 3.412, *p* < 0.018).

Figure 7C plots the average preference ratings for the grayscale, thresholded and edges only images as a function of the corresponding perceived complexity of these images. In all three cases one can see an inverted U-shaped function with the preference peaking for the images with the intermediate perceived complexity.

#### Interindividual Differences in Preference for Fractal Images

**Figure 8:**
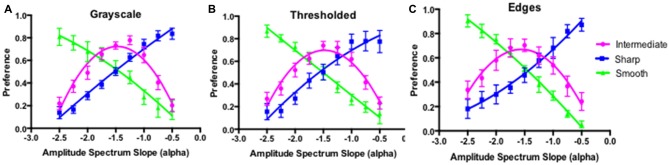
**Summary preference function for participants subjectively classified as belonging to different preference sub-groups for the grayscale (A), thresholded (B) and edges only (C) images in Experiment 1**.

Although all three different image types exhibited the “universal” pattern of preference for intermediate amplitude spectrum slopes, in each case there were clearly noticeable differences between individual preference functions of different observers. Thus, for each of the three image types we subjectively defined distinct sub-groups of preference functions as illustrated in Figure 8. Over 90% of participants could be easily classified as exhibiting three readily distinguishable and distinct patterns of preference. We label these three patterns as “intermediate”, “smooth”, and “sharp” in line with the appearance of the image in each category that was associated with the peak preference. The “intermediate” group exhibits a typical peak preference for images with the intermediate amplitude spectrum slope values, while the “smooth” and “sharp” groups consist of participants with the peak preference for the images with the steep (blurry in appearance) and shallow (sharp in appearance) amplitude spectrum slope values respectively. Participants in the “smooth” group exhibit a linear decrease in preference as a function of the amplitude spectrum slope while those in the “sharp” group exhibit a linear increase in preference.

The panels (A) to (C) in Figure 8 plot the average preference function for each of the three subgroups with grayscale, thresholded and edges only images respectively. The data points are the average preference functions of the each subgroup with the 95% CI and the corresponding second-order polynomial fits. The highest number of participants, approximately 50% across all image types was subjectively classified as the intermediate group, followed by approximately 20% of participants in each of the smooth and sharp groups. Approximately 10% of participants exhibiting either flat, or noisy pattern of preference were classified as the “other” group (data not shown).

**Figure 9:**
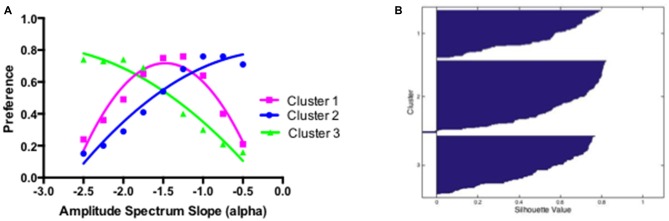
**(A)** Summary preference function for participants classified as belonging to one of the three clusters determined by the k-means clustering procedure. **(B)** *Silhouette* values for participants assigned to different clusters.

These subjectively derived groups were confirmed by the k-means clustering approach performed on a 9 × 278 matrix of individual preference scores for the grayscale, thresholded and edges only images. K-means clustering was performed with the MATLAB *k-means* function utilizing the default squared Euclidean distance measure. Participants were clustered into three subgroups and the visual preferences for participants when assigned to these cluster solutions are plotted in Figure 9A. For each identified cluster, the average *silhouette* values, representing how well each participant fits within the assigned cluster, were calculated with the *silhouette* MATLAB function and shown in Figure 9B. The average *silhouette* value of all participants for a three-cluster solution was 0.56 (95% CI from 0.53 to 0.58) with means for Clusters 1, 2 and 3 of 0.51, 0.60 and 0.52 respectively. Overall, the three identified clusters seem to provide a good partitioning of individual preference functions (Rousseeuw, 1987). In addition, the clusters derived via the k-means procedure seem to correspond remarkably well with the subjective classification of individual preference functions into intermediate, smooth and sharp groups respectively.

#### Dimensional Structure of Interindividual Differences

**Figure 10:**
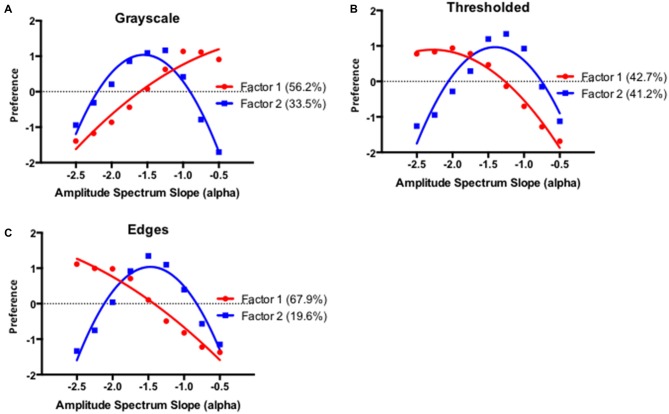
**Summary preference functions for the two factors resulting from the Q-mode principal component analysis for the grayscale (A), thresholded (B), and edges only images (C).** Percentages in each panel indicate the proportion of variance explained by each factor.

In order to investigate the dimensional structure of the observed interindividual differences we performed a Q-mode factor analysis as was done in McManus (1980) and McManus et al. (2010). Q-mode analysis is based on a transposed data set and pairwise correlations between the participants instead of stimuli. The principal component analysis identified two factors that were able to explain more than 80% of variations between observers for each image type. Summary preference functions for the two factors with each image type are depicted in Figure 10.

Factor 1 differentiates between preference for images with either high or shallow amplitude spectrum slope values and it explains 56.2%, 42.7%, and 67.9% of the variance for the grayscale, thresholded and edges only images respectively. With the grayscale images this factor has positive and negative loadings associated with the lower and higher amplitude spectrum slope values respectively while the opposite seems to be the case with the threshoded and edges only images. As discussed previously, the differences in Factor 1 loadings between the grayscale and other images are consistent with the specific effect of image blur in grayscale images. Factor 2 can be described as the intermediate preference factor and it explains 33.5%, 41.2% and 19.6% of the variance for the grayscale, thresholded and edges only images respectively. Overall, these results suggest that remarkably similar, image driven, structural dimensions mediate inter individual differences in the preference for the range of visually distinct images with fractal-like scaling characteristics.

## Experiment 2: Stability of Interindividual Differences in Preference for Fractal Scaling Characteristics Across Variety of Patterns

In Experiment 1, we identified three major sub-groups of individual preference functions in grayscale, thresholded and edges only fractal images. The patterns of individual preference functions in different sub-groups and the dimensional structure of the observed inter individual differences were remarkably similar across different image types. However, the observed similarity concerns the regularity between different groups of observers. The main aim of this study is to investigate the extent to which the patterns of preferences across different stimulus categories remain “stable” at the level of individual observers.

The individual preferences have been reported as generally consistent and stable over time. For example, Vessel and Rubin (2010) reported high within-observer split-half reliability of individual observers’ preferences for both abstract and real-world images (0.67 and 0.70 respectively). Even when the testing occasions were separated by longer intervals in between, such as days or weeks, the high level of consistency in individual preference was still evident (Halpern et al., 2008; McManus et al., 2010). However, these measures of stability are generally based on the same type of stimuli.

In this study, we probe whether participants with preference for certain fractal structure will also prefer similar structure in a range of visually distinct images. In other words, stability here denotes the consistency of an individual’s preference patterns. For example, an individual whose preference for high amplitude slope is maintained for all fractal classes would be regarded as more stable than an individual whose preference for high amplitude slopes is limited to only one class. Stable patterns of individual differences, or a lack thereof, can suggest factors mediating preference for different kinds of fractal classes and variations in fractal scaling characteristics of images.

**Figure 11:**
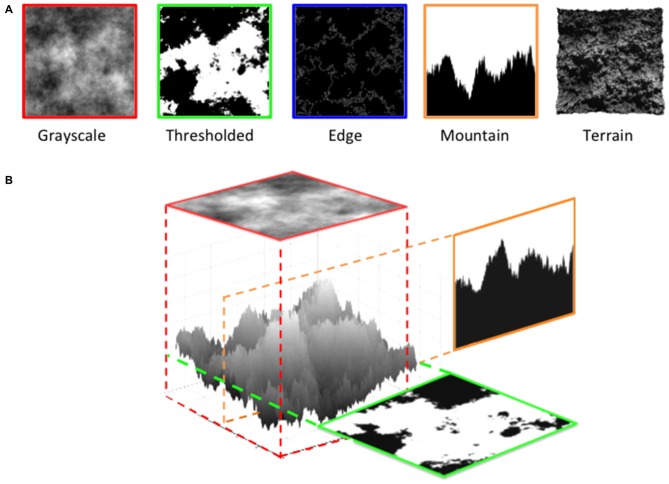
**Types of images with fractal-like scaling characteristics used in Experiment 2. (A)** From left to right, examples of grayscale, thresholded, edges, mountain and terrain images. **(B)** An illustration of the relationship between different types of image, all derived from the original seed grayscale image.

For this purpose, we measure group and individual preference functions in the same group of observers across different image types. Figure 11A shows examples of image types used in this study, that in addition to the grayscale, thresholded and edges only images include “mountain” and “terrain” derivations from the original grayscale image. As it can be seen in Figure 11B, the terrain image is simply a depth plot of the grayscale image in which the height of each point is proportional to the grayscale value (brightness) of the corresponding point in the grayscale image. The “mountain” variant is created by taking a vertical slice through the terrain to create 1D mountain profile. In relation to the terrain image, the thresholded variant can also be viewed as a horizontal slice taken through the terrain at a selected height. Then all of the terrain below this height is colored black and all of the terrain above is colored white. In fractal terminology, this is often referred to as the coastline pattern (black being the water), with the “edges only” image generated from this image by highlighting the coastline edges in white.

Taken together, these five families of fractals are powerful stimuli for examining people’s generic responses because, although superficially quite different in appearance, they all possess identical scaling properties. Based on our previous investigations, we expect that preference within each image type will vary systematically as a function of amplitude slope. Stability in preference will be assessed by calculating correlation coefficients between preference scores for different image types for each individual observer.

### Experiment 2: Materials and Methods

#### Participants

A total of 50 Psychology undergraduate students at University of New South Wales (mean age 19.28; 50% male) participated in exchange for course credit. All observers viewed all five different image types. Informed consent, testing and debriefing procedures were approved by the UNSW Human Research Ethics Advisory Panel.

#### Design

This study employed a 5 (fractal image type: grayscale, thresholded, edges, mountain, and terrain) × 9 (amplitude spectrum slope: from 0.5 to 2.5 in increments of 0.25) repeated measures design.

#### Stimuli

**Figure 12:**
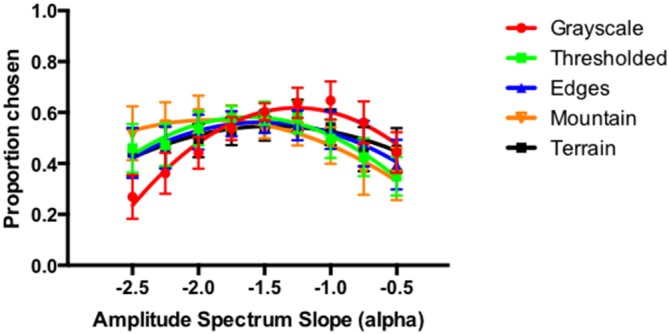
**Group preference functions for different type of fractal images in Experiment 2 (*N* = 50)**.

The grayscale, thresholded and edges only images were constructed in the same way as described in Experiment 1. The terrain images were created by converting the grayscale images into a depth map by using a 3D Adobe Photoshop tool which creates a mesh with the height of each point proportional to the grayscale value of the corresponding point in the grayscale image (see Figure 12). The “mountain” images were created by taking a vertical slice through the terrain to create 1D, black and white mountain profile. Each series of images contained nine experimental images varying in the input amplitude spectrum slope α values of 0.5, 0.75, 1.00, 1.25, 1.50, 1.75, 2.00, 2.25 and 2.50 of the original grayscale images. For each different image type five different separate sets were created and as in Experiment 1 the mean brightness and the RMS contrast of all images were controlled at 126 and 0.30 respectively.

#### Apparatus

The same apparatus as in Experiment 1 was used.

#### Procedure

As in Experiment 1, the paired comparison procedure was used to measure preference in five different types of images. Each participant viewed 360 unique pairings of different amplitude slope value across five different image types. As mentioned before there were five different sets with different seed images and they were counterbalanced across different participants such that each set was viewed by a subgroup of 10 participants.

### Experiment 2: Results and Discussion

#### Average (Group) Preference for Different Types of Images

**Figure 13:**
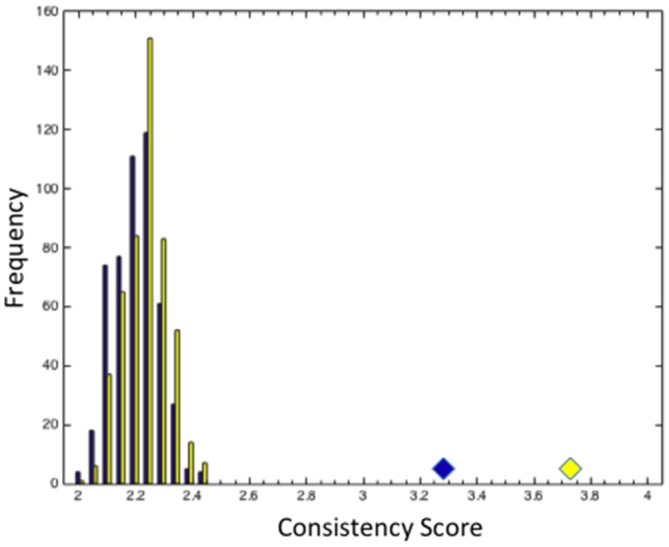
**The distribution of consistency scores for 500 bootstrap simulations with randomly perturbed sub-group (yellow columns) or cluster (blue columns) membership.** Blue and yellow diamond represent the average consistency scores for subjectively defined sub-groups and for clusters assigned by k-means analysis.

The average visual preference functions for the five different types of images are depicted in Figure 13. The preference expressed as the proportion of trials on which the certain image was chosen plotted as a function of the input amplitude spectrum slope of the original grayscale images. The error bars correspond to 95% CI associated with the respective condition means. As before, the average preference functions show a similar pattern with the visual preference highest for the intermediate amplitude spectrum slopes or fractal dimension values for the grayscale, thresholded, edge and terrain images. With the mountain images, the average preference function, while showing the same trend as other images at the shallower amplitude spectrum slope values, remained high for the intermediate as well as the higher amplitude spectrum slope values. Repeated-measures ANOVA revealed the significant main effect of the amplitude slope (*F*_(8,42)_ = 5.866, *p* < 0.000) and significant interaction between amplitude slope/fractal dimension (*F*_(32,18)_ = 2.262, *p* < 0.035). The main effect of image type was not significant (*F*_(4,46)_ = 1.676, *p* < 0.172).

#### Stability of Inter-individual Differences in Preference for Fractal Images

Like in the Experiment 1, it was possible to subjectively identify four distinct sub-groups of individual preference functions with each of five different image types. On average, across all image types, the percentage of participants classified as the intermediate, sharp, smooth and other sub-groups were 32.4%, 26.8%, 31.2% and 9.6% respectively. Similarly, the, k-means clustering procedure yielded 21.2%, 34%, 34.4% and 10.4% of participants classified as intermediate, sharp, smooth and other groups respectively. A Chi-square analysis has revealed a significant difference between the frequency distributions of different sub-groups across different image types (35.81, *p* < 0.0003 for subjectively classified and 25.12, *p* < 0.05 for the k-means clustering). There was relatively higher proportion of intermediate and sharp categories with the grayscale and terrain images compared to the thresholded, edge and mountain images. While these variations appear to be somewhat larger than those observed in Experiment 1, it is hard to make the direct comparisons and conclusive interpretations of these differences given the variations in the experimental design between the two studies.

The within subject design employed in this study allows us to investigate intraindividual stability of preference for variations in fractal scaling across a widely different image types. As an index of the stability of individual preference functions, for each observer, we compute the consistency score for each participant by counting the highest number of consistent response types across the five image categories. The consistency scores could range from minimum of two to the maximum of five with the obtained average consistency scores of 3.8 (95% CI from 3.5 to 4.1) and 3.32 (95% CI from 3.0 to 3.6) for the subjectively- and k-means clustering-based classifications. In order to estimate the baseline consistency score we performed random permutations of assigned classifications within each image type and re-calculated consistency scores for each individual participant. Figure 13 depicts the distribution of average consistency scores based on 500 bootstrap simulations of random permutations for subjectively- (yellow) and k-means clustering- (blue) based classifications. The yellow and blue symbols represent the obtained average consistency scores based on the two classification procedures respectively.

**Figure 14:**
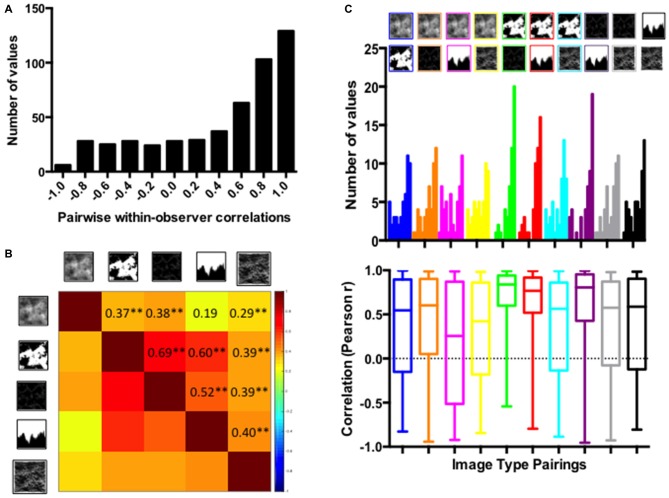
**Pairwise, within-observer, correlations between different image type: (A)** Histogram of individual correlations between preference functions for different image types (*N* = 500); **(B)** Heatmap depicting the average individual correlations between different image pairings (**p < 0.001). **(C)** Pairwise, within-observer, correlations between different image types broken by the specific image type pairings: (top panel) Histogram of individual correlations for each specific image type pairing; (bottom panel). The corresponding box and whisker plots of correlation coefficients of individual preference functions between different image type pairings.

As another index of the stability of individual preference functions, we calculate pairwise correlations between participant’s preference scores among different types of images. With five different types of images, there were 10 unique pairings for each of 50 observers, resulting in a total of 500 individual correlation coefficients; 50 for each of the ten unique pairings between different image types. Figure 14 shows the histogram of all 500 correlations across all individual and all image types (panel A) and the average correlation between preference scores for different image type parings for 50 observers (panel B). Figure 14C shows the histograms of intraindividual correlations for each of the specific image type pairings (top panel) and the corresponding box and whiskers plots of the frequency distributions of these intra-individual correlations. In the boxplot, the 25–75 percent quartiles of the distribution are drawn using a box. The median is shown with a horizontal line inside the box. Maximum “whisker” length corresponds approximately to ±2.7 SD and 99.3% coverage.

As the various panels in Figure 14 show, the levels of within-observer consistency in preference between different image types observed in this study are quite high despite the substantial differences in the appearance of these images. As can be seen in Figure 14B, the highest individual correlations of preference scores were obtained between the thresholded and edges only images (Mean = 0.69; 95% CI = 0.56–0.78, *t*_(49)_ = 11.95, *p* < 0.0001), with relatively high intraindividual correlations also obtained between threshoded and mountain (Mean = 0.60; 95% CI = 0.45–0.73, *t*_(49)_ = 8.658, *p* < 0.0001) as well as edge and mountain images (Mean = 0.52; 95% CI = 0.31–0.68, *t*_(49)_ = 5.458, *p* < 0.0001). The lowest intraindividual correlations were obtained between the grayscale and other types of images, in particular between the grayscale and mountain images (Mean = 0.19; 95% CI = −0.02 to 0.37, *t*_(49)_ = 1.756, *p* = 0.0853).

In general, although the observed within-observer consistency does not reach the levels of the immediate and short-term (30 min) test-retest reliability reported by McManus et al. (2010), the highest levels of within-observer consistency observed in this study are similar to the measures of split-half reliability in aesthetic preference for the abstract and real world images reported by Vessel and Rubin (2010). Given the large heterogeneity of image types used in our study, the observed levels of within-observer consistency can be considered as measure of generalizability of aesthetic preference across different image types, as opposed to a more traditional conceptualization of test-retest, or split-half reliability.

The generalizability seems the highest between thresholded, edge and mountain images and the lowest between the grayscale and mountain and grayscale and terrain images. While the preference scores seem to be overwhelmingly driven by the variations in the fractal scaling characteristics across all image types, we think that relatively lower agreements in the preference scores between specific pairings are attributable to the presence of additional image features known to be effective in influencing image preference. For example, in the case of grayscale and mountain images we believe that image blur and contour smoothness respectively have influenced the respective individual preference functions (Bar and Neta, 2006; Juricevic et al., 2010).

We believe that might be related to the high degree of perceived image blur at higher amplitude spectrum slope values in grayscale images, which is not present in thresholded, edges only and mountain images. Previous studies, including our own, have reported decreased visual preference as a function of image blur (Juricevic et al., 2010; Spehar and Taylor, 2013; Spehar et al., 2015). The thresholded, edges only and mountain images are not associated with any degree of edge blur and at certain levels are comprised of smoothly curved contours. The smooth contour curvature is a feature often associated with a high degree of visual preference and aesthetic rating (Hogarth, 1753; Bar and Neta, 2006; Carbon, 2010; Bertamini et al., 2015; Gómez-Puerto et al., 2016) and this might have contributed to the intraindividual differences observed in our study.

#### Dimensional Structure of Inter-individual Differences

**Figure 15:**
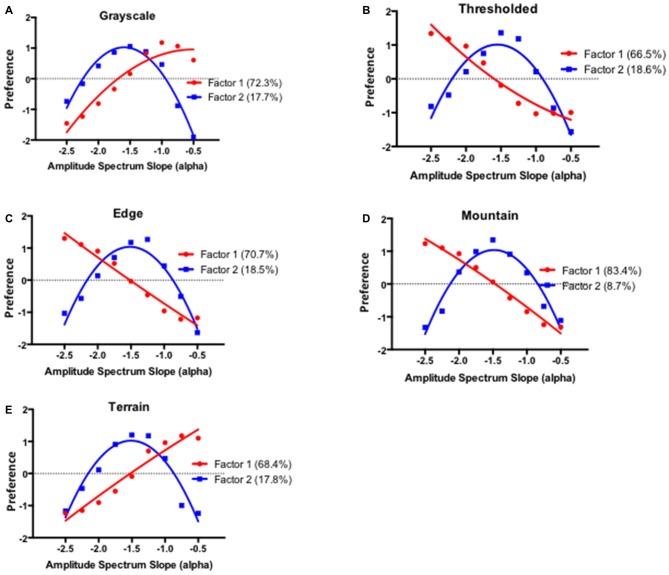
**Summary preference functions for the two factors resulting from the Q-mode principal component analysis for the grayscale (A), thresholded (B), edges (C), mountain (D) and terrain images (E) respectively.** Percentages in each panel indicate the proportion of variance explained by each factor.

Like in Experiment 1, in order to investigate the dimensional structure of the observed inter-individual differences we performed a Q-mode factor analysis (McManus, 1980; McManus et al., 2010) on pairwise correlations between preference scores of different observers. Again, the principal component analysis identified two factors that were able to explain a large proportion of variations between observers for all image types. The summary preference functions for different image types for the two factors are illustrated in Figure 15.

The Factor 1 accounts for approximately 72% of the total variance across different image types and, like before, differentiates between preferences for images with either low or high amplitude spectrum slope values. The Factor 1 loadings for the grayscale and terrain images are positive with the low amplitude spectrum slope values and negative with the high amplitude spectrum slope values, suggesting the similar effect of image blur and surface smoothness on visual preference between different observers. The opposite pattern holds for the Factor 1 loadings with the grayscale, edge and mountain images where contour smoothness is a likely factor affecting visual preference at high amplitude spectrum values in some observers. Factor 2 accounts on average for approximately 16% of the total variance and corresponds to the preference for the intermediate spectrum slope values.

## General Discussion

Our study extends the previous efforts to systematically investigate both the extent and structure of individual differences in aesthetic preference (Thorndike, 1917; McManus, 1980; Höfel and Jacobsen, 2003; McManus et al., 2010; Vessel and Rubin, 2010). We focus on individual differences in preference for variations in fractal-like scaling characteristics in visual patterns ranging from filtered synthetic noise to fractal contours and terrain images and find that the average group preferences with all image types mostly conform to the previously reported pattern of highest preferences for the intermediate levels of fractal-like scaling characteristics (Spehar et al., 2003, 2015; Juricevic et al., 2010; Spehar and Taylor, 2013). Our analyses also suggest that the fractal edge parameters, not the overall photometric characteristics, are important drivers of both image preference and perceived complexity in these images.

Both the subjective and statistical clustering analysis of the observed interindividual differences revealed distinct subgroups that were highly stable across all image types. While a sizable proportion of participants exhibited a typical peak preference for the intermediate fractal-scaling characteristics, other participants exhibited either a linear increase in preference with increasing amplitude spectrum slope, or a linear decrease in preference with increasing amplitude spectrum slope. Labeled “smooth” and “sharp” respectively, these sub-groups had approximately 20% of participants each in Experiment 1 and around 30% of participants in Experiment 2. The patterns of preference exhibited by these two groups in our study are quite similar to what was observed in a recent study by Satgunam et al. (2013) investigating factors affecting preference for enhanced video quality. They measured visual preference for videos with different levels of contour enhancement levels, resulting in contours with different degrees of sharpness. Although they were not interested in individual differences at the outset of their study, they found that participants could be clearly classified into those who preferred contour enhancement (also labeled “sharp”) and those who preferred unenhanced images (also labeled “smooth”). As the increasing levels of contour sharpness are associated with the concomitant changes in the amplitude spectrum slopes of the entire image and the associated fractal scaling characteristics, the preference for different degrees of contour enhancement might reflect the preference for the images with varying fractal scaling characteristics, as observed in our study.

Our investigation of aesthetic preferences for fractal-like scaling characteristics was extended to include 1D contour images (mountain profiles) as well as the simulated three-dimensional terrain images. Moreover, the visual preference across different image types was measured in the same group of participants, allowing us to systematically investigate the stability of individual preferences for fractal-like scaling characteristics. Both the average and individual preference functions were correlated across different image types, thus demonstrating a good agreement and stability of image-driven influences on visual preference. Individual observers demonstrated high levels of consistency in preference for certain fractal scaling characteristics across a large range of image categories. In addition, a formal principal component analysis (Q-mode) has revealed a highly consistent latent dimensional structure of the observed interindividual differences in both experiments. For all investigated image types, the principal component analysis identified two factors that were able to explain more than 80% of variations between observers.

These findings are in agreement with the past finding that individuals tend to be consistent in the particular dimension or structure of stimuli they preferred (Jacobsen, 2004; Halpern et al., 2008; McManus et al., 2010; Vessel and Rubin, 2010). However, our measures of stability extend the traditional split half or test-retest measures as they speak to the generalizabiliy of preference patterns across images types ranging from one-dimensional contours to the simulated three-dimensional surfaces.

Abstract images have low semantic meaning, and we do not believe that the observed patterns of preference were substantially influenced by the factors such as interpretability and shared semantic associations related to these images (Vessel and Rubin, 2010). Our initial experiments (Spehar et al., 2003) used stimuli for which we couldn’t exclude the possibility of a semantics playing a role—photographs of natural objects such as trees and clouds, Pollock paintings which are frequently referred to as organic and look to many like trees and vegetation, and computer simulations of clouds (which looked like clouds!!!). Although Rogowitz and Voss (1990) suggested that participants could derive representational meaning even from abstract images, such as those resembling clouds, the physical appearances of stimuli used in the present study varied substantially, making it unlikely that participants derived similar representational meaning across different image types. Semantic meaning is consequently unlikely to be the essential factor for the preference stability found in the present study.

Another possible factor that could facilitate the stability of preferences are the properties inherent and shared in stimuli. Güçlütürk et al. (2016) have recently reported systematic differences in visual preference for patterns varying in perceived and objectively measured levels of complexity. All our images possess scale invariant characteristics that are encountered in natural scenes. Our visual system has evolved within, and has the enhanced processing capabilities related to these characteristics (Knill et al., 1990; Tadmor and Tolhurst, 1994; Párraga et al., 2000, 2005; Hansen and Hess, 2006). Consistent with these notions is our own work showing a close association between the aesthetic judgments of spatial patterns and visual sensitivity to precisely defined spatial structure of such patterns (Spehar et al., 2015).

## Author Contributions

BS and RPT designed the study, analyzed and interpreted the data and wrote the article. NW was involved in running of experiments, data collection and data analysis.

## Conflict of Interest Statement

The authors declare that the research was conducted in the absence of any commercial or financial relationships that could be construed as a potential conflict of interest.

## Footnotes

^1^https://github.com/vistalab/vistadisp/tree/master/exptTools2/noiseTools
